# Alcohol-induced risk taking on the BART mediates alcohol priming

**DOI:** 10.1007/s00213-013-3377-1

**Published:** 2013-12-13

**Authors:** Abigail K. Rose, Andrew Jones, Natasha Clarke, Paul Christiansen

**Affiliations:** School of Psychology, University of Liverpool, 2.32 Eleanor Rathbone Building, Bedford Street South, Liverpool, L69 7ZA UK

**Keywords:** Alcohol, BART, Risk taking, Intoxication, Priming, Urge, Social drinkers

## Abstract

**Rationale:**

Hazardous drinking has been associated with risk taking and alcohol priming effects. However, the potential relationship between risk taking and priming has not been investigated. The Balloon Analogue Risk Task (BART) is a behavioural measure of risk taking which appears to be associated with drinking behaviour. However, alcohol's acute effects on BART performance are not clear, and the potentially mediating effect of alcohol-induced risk taking on priming has not been tested.

**Objectives:**

To assess the effects of a priming dose of alcohol on BART performance; to determine the predictive utility of the BART on drinking habits; and to identify whether alcohol-induced risk taking mediates alcohol priming (urge to drink).

**Methods:**

A total of 142 participants provided data on drinking habits and trait-like impulsivity and sensation seeking. The BART was then completed after consuming alcohol (0.6 g/kg) or placebo (between-subjects design). Baseline and post-drink measures of alcohol urge were also taken.

**Results:**

Alcohol consumption increased urge to drink (priming) and risk taking on the BART. In the alcohol group only, risk taking on the BART predicted unique variance in weekly alcohol consumption and bingeing. Mediation analysis showed that risk taking following alcohol consumption mediated alcohol priming.

**Conclusions:**

This is the first study to show that alcohol acutely increases risk taking on the BART. Results suggest that social drinkers susceptible to alcohol-induced risk taking may be more likely to drink excessively, perhaps due to increased urge to drink (priming).

**Electronic supplementary material:**

The online version of this article (doi:10.1007/s00213-013-3377-1) contains supplementary material, which is available to authorized users.

## Introduction

The link between addictive behaviours and impulsivity has been well documented (Jentsch and Taylor 1999; Winstanley et al. 2010). However, impulsivity is a broad construct which covers several aspects (e.g., risk taking, inhibitory control, delay discounting) which may be differentially related to drinking. In order to better understand the mechanisms underlying drinking behaviour, it is important to identify which of these aspects of impulsivity are most relevant. For example, hazardous drinkers display greater discounting of delayed rewards and impaired inhibitory control (Christiansen et al. 2012a, b; Murphy and Garavan 2011), while acute doses of alcohol can impair inhibitory control in social drinkers (Rose and Duka 2008). In addition, binge drinking is associated with increases in risky behaviour, including unprotected sex in college students (Patrick 2013), while dependent drinkers demonstrate impaired inhibitory control on a range of tasks (Jentsch and Taylor 1999). Together, these findings suggest that alcohol increases impulsive behaviour; however, there appears to be a reciprocal causal relationship between drinking and aspects of impulsivity.

Trait-like impulsivity (measured by Barratt's Impulsivity Scale [BIS], which covers aspects of attentional, motor, and non-planning impulsivity) has been positively associated with bingeing in university students (Kazemi et al. 2011) and risky behaviour in dependent drinkers when intoxicated (Jakubczyk et al. 2013). In alcohol dependent adolescents, impulse control disorders (e.g., attention deficit hyperactivity disorder [ADHD) have been found to precede alcohol use (Kuperman et al. 2001). A cohort study of young adolescents found that, after controlling for baseline alcohol use, increases in sensation seeking and risk taking predicted increases in alcohol consumption (MacPherson et al. 2010).

Sensation seeking (SS) is a personality characteristic which has been identified as a risk factor for problem drinking (Windle et al. 2008), and is the tendency to seek out novel, varied, and intense experiences, and the willingness to make social, personal, and financial risks for such experiences (Zuckerman and Kuhlman 2000). Risk taking covers behaviours which are primarily rewarding but which may also result in harmful consequences to the individual or others (e.g., unprotected sex) (MacPherson et al. 2010), and is an aspect of impulsivity which has been particularly related to drinking (Claus and Hutchison 2012). Indeed hazardous drinking is a type of risk taking; it can result in many positive outcomes such as sociability, feeling stimulated or less stressed, but a number of negative consequences are also possible such as hangovers, accidents, and aggression.

The computer-based Balloon Analogue Risk Task (BART) was developed as a behavioural risk taking task (Lejuez et al. 2002) in which the participant can respond to inflate a balloon. Each response inflates the balloon and each successful inflation is associated with winning points or money; the larger the balloon the greater the reward. However, each inflation is associated with the risk that the balloon will burst and all points/money earned for that balloon will be lost. In adults, Lejuez et al. (2002) found that BART risk taking correlated with self-reports of trait-like impulsivity (including BIS and SS) and accounted for unique variance in risky behaviours (alcohol, nicotine and illicit substance use, gambling, unprotected sex). Positive correlations between the BART and risky behaviours, but not impulsivity traits, have also been found in adolescents (Lejuez et al. 2003). Weafer et al. (2011) found a positive association between the BART and drinking frequency in social drinkers, and a trend for an association between the BART and quantity of alcohol consumption in social drinkers with and without ADHD. Weafer et al. (2011) did not control for BIS or SS so it is unclear whether the BART is useful in identifying a unique relationship between aspects of impulsivity and drinking beyond that of the more traditional self-report measures. However, Fernie et al. (2010) did control for trait-like impulsivity and gender, and found that the BART predicted alcohol use. Importantly, Fernie et al. (2010) did not find a predictive relationship between other behavioural tasks which assess non-risk taking aspects of impulsivity (Go/No-Go, Stop signal, and Delay Discounting) and drinking, supporting the suggestion that risk taking may have better predictive utility than other components of impulsivity (Claus and Hutchison 2012). This is also supported by the finding that, in adolescents, the BART (but not self-reported BIS or SS) correlates with and predicts substance use (Aklin et al. 2005). Although the link between BART and risky behaviour in adolescents has been confirmed several times, evidence suggests that BART performance can become less risky over trial blocks (Lejuez et al. 2005; Lejuez et al. 2007). It is unclear how long potential practice effects last, so the current study employed a between subject design.

It is important to remember that aspects of impulsivity display transient changes, and it has been suggested that in addition to trait-like impulsivity, transient increases in relevant aspects may influence drinking (Rose and Grunsell 2008). Although acute alcohol consumption (0.6 g/kg) has been shown to impair inhibitory control aspects of impulsivity (e.g., motoric and interference inhibition) (Rose and Duka 2008), very few studies have assessed alcohol's acute effect on BART risk taking. Reynolds and colleagues (2006) found no effect of a 0.4 or 0.8 g/kg dose of alcohol; however, this study required completion of five different tasks 15–105 min after alcohol; alcohol effects may have been masked by fatigue or the impact of different blood alcohol levels at time of testing. Given the reciprocal causal relationship between drinking and aspects of impulsivity (including risk taking), and the findings that BART is associated with substance use (Lejuez et al. 2002; 2003), it is necessary to clarify alcohol's acute effects on the BART without these potential confounds.

Theoretically, it has been suggested that alcohol impairs our ability to control our behaviour which can increase risk taking, which includes drinking more (e.g., bingeing) (Lane et al. 2004). However, another key mechanism underlying drinking is 'alcohol priming', which refers to the finding that moderate consumption (~0.6 g/kg alcohol) can increase craving and drinking (de Wit and Chutuape 1993; Rose and Duka 2006), and may be associated with harmful drinking (e.g., bingeing; Rose and Grunsell 2008). Traditionally, priming effects have been associated with alcohol's ability to increase appetitive responses to alcohol (e.g., initial consumption enhances the positive reinforcing effects of drinking) (Rose and Duka 2006), and research into priming and impulsive alcohol effects have been investigated separately. However, it has recently been suggested that appetitive responses are able to influence drinking behaviour *because* alcohol acutely increases aspects of impulsivity which may impair our inhibition of appetitive responses (Field et al. 2010). The possibility that alcohol's augmentation of aspects of impulsivity mediates alcohol priming is interesting but has yet to be investigated. Given that alcohol priming may be associated with hazardous drinking and that hazardous drinking is a type of risk taking, we would argue that alcohol-induced risk taking may be one aspect of impulsivity that might mediate alcohol priming.

The primary aims of the current paper were to (1) identify the effect of a priming dose of alcohol on risk taking (BART), (2) assess whether alcohol-induced and sober risk taking predicts drinking, and (3) determine whether alcohol-induced risk taking mediates alcohol priming (urge to drink). We hypothesised that (1) a priming dose of alcohol would increase risk taking; (2) both alcohol-induced and sober BART performance would predict unique variance in drinking habits (while controlling for impulsive traits); and (3) alcohol-induced risk taking would mediate the alcohol priming effect.

Given that research has found different relationships between aspects of impulsivity (e.g., self-report BIS, SS, and behavioural BART), and between aspects of impulsivity and drinking behaviour, we also conducted secondary analysis which showed that (1) intoxicated but not sober BART performance correlated with drinking behaviour, (2) only sober BART performance correlated with self-reported SS, and (3) results were not affected by gender (see Supplemental document for analysis and discussion).

## Methods

### Participants

One hundred and forty two participants were recruited (67 men) from the student population of the University of Liverpool. Participants were required to consume alcohol on a weekly basis, be in good general health, and speak fluent English. Exclusion criteria included a past or present alcohol or drug use disorder, and being on medication which could interact with alcohol. The study obtained ethical approval from the University of Liverpool's Ethics Committee.

### Self-reported drinking habits

*Alcohol Use Disorders Identification Test* (AUDIT) (Saunders et al. 1993) identifies the hazardous and harmful patterns of alcohol use. The AUDIT has ten items (score 0–4), and shows good internal consistency as a single factor when used in college students (Cronbach's *α* = 0.82) (Shields et al. 2004).

*Time Line Follow Back* (TLFB) (Sobell and Sobell 1992) assesses typical weekly alcohol consumption (unit consumption [1 UK unit = 8 g alcohol]) and binge frequency (female: ≥6 units p/drink episode, males: ≥8 units p/drink episode) using a 2-week diary format. The 2-week TLFB has been used in previous laboratory/priming studies (Christiansen et al. 2012b) to reliably assess university students' self-reported drinking behaviour.

### Self-reported alcohol urge

*Alcohol Urge Questionnaire* (AUQ) (Bohn et al. 1995) is used to measure current alcohol urge across three domains: desire for alcohol, expectation of positive effect from drinking, and inability to avoid drinking if alcohol was available. The AUQ produces a single alcohol urge factor which shows good internal consistency (*α* = 0.86) (MacKillop 2006).

### Self-reported impulsive characteristics

*Barratt's Impulsivity Scale* (v1) (Patton et al. 1995) assesses trait-like impulsivity across three dimensions: attentional, motor, and non-planning. However, we used the BIS total score as it has the best internal consistency (*α* = 0.83) (Stanford et al. 2009). The BIS consists of 30 items with higher scores indicating greater impulsivity.

*Sensation Seeking Scale* (SSS) (Zuckerman et al. 1993) measures impulsive SS from the 19 item set taken from the Zuckerman–Kuhlman Personality Questionnaire. Items focus on a lack of planning and acting without thinking, and behaving for 'experience seeking', and a willingness to take risks for excitement and novelty. Higher scores indicating greater SS (*α* = 0.80) (Stanford et al. 2009), which has been associated with risk taking behaviours (Zuckerman and Kuhlman 2000).

### Behavioural risk taking

*Balloon Analogue Risk Task* (Lejuez et al. 2002). During the BART, participants used mouse clicks to pump up a simulated balloon. Each time the participant pumped the balloon they accrued $0.05 in a temporary bank (hypothetical reward), which was visible at all times. With each pump, the balloon grew slightly larger on the screen. Participants were cautioned that the balloon could burst at any point from the first pump onwards. They were also informed that, at any time, they could transfer the hypothetical money in their temporary bank to a permanent bank (also visible at all times) by clicking a box labelled 'Collect $$$'; however, if the balloon burst they would lose all the money in their temporary bank. Following an explosion or collection, the size of the balloon was reset and the temporary bank returned to $0. The balloons were set to explode on a variable ratio, with the average explosion point of 64 pumps (for detailed information, see Lejuez et al. 2003). Each participant pumped up 20 balloons (i.e., trials). Based on previous research (e.g., Lejuez et al. 2002), we used 'Adjusted average pumps' (the average number of pumps on balloons that did not explode) as the main outcome variable.

### Procedure

Testing sessions took place between 12 pm and 6 pm in a laboratory in the School of Psychology. Participants were asked to consume a high carbohydrate, low fat meal the night before and a light meal (e.g., a sandwich) an hour before the experimental session to help balance alcohol absorption and metabolism across participants. Participants were asked to avoid drinking alcohol before the experiment, and to avoid heavy drinking the night before. Participants attended the laboratory for one experimental session, and were randomised to the alcohol or placebo group (stratified by gender). After providing written informed consent, participants were weighed and a 0.0 mg/l breathalyser was required for testing to start.

Participants completed the drinking habit and impulsivity self-reports (AUDIT, TLFB, BIS, SSS), and baseline alcohol urge (AUQ), before consuming the experimental drinks. Based on previous priming research, a moderate dose of alcohol (0.6 g/kg) was given as vodka mixed with lemonade to provide a 400 ml beverage (Rose and Duka 2006). The placebo was 400 ml of lemonade with a vodka mist sprayed over the glasses. The beverage was divided into three equal drinks, and participants were given 6.5 min to consume each beverage (semi-structured consumption). Following consumption, participants rested for 20 min to ensure testing took place during the ascending portion of the blood alcohol curve (Rose and Duka 2006). Participants provided a breathalyser reading before completing a second AUQ followed by the BART. A final breathalyser reading was taken before participants were debriefed.

## Results

### Sample characteristics

Participants were aged 20.33 (SD, ±3.74) years. Average weekly alcohol unit (1 unit = 8 g alcohol) consumption was 22.92 (±15.26) and weekly binge frequency was 1.45 (±0.94). Mean AUDIT score was 15.17 (±5.57) which is above the cut-off for hazardous drinking (≥8). In terms of personality characteristics, participants scored 67.18 (±10.68) on the BIS and 49.08 (±8.73) on the SSS. Groups were well matched and did not differ on these factors (*p* > 0.1).

### Breath alcohol concentration (BrAC)

BrAC readings before and after the task were 0.36 (±0.10) and 0.36 (±0.08) mg/l, respectively.

### Alcohol priming (urge to drink)

**Fig. 1:**
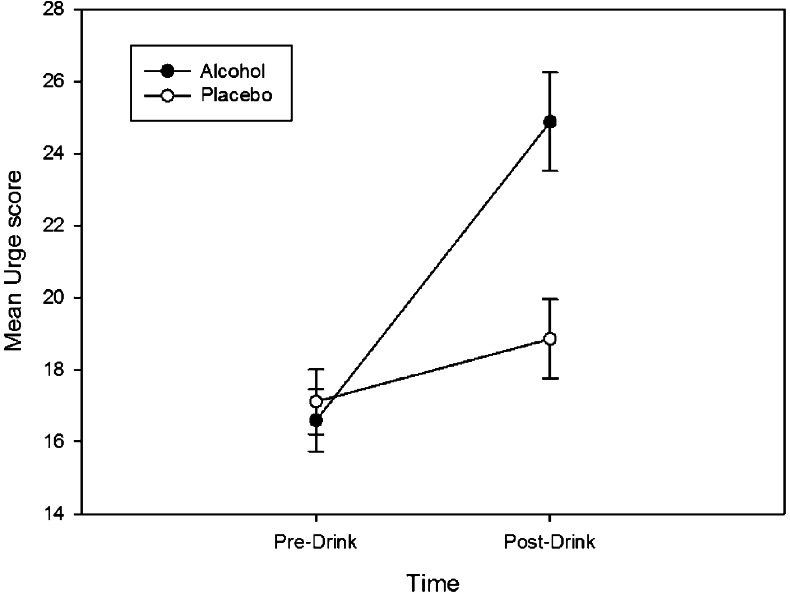
Alcohol urge increased in both conditions but this effect was greater after alcohol, relative to placebo, consumption

*Alcohol Urge* (see Fig. 1): a 2 (group: alcohol/placebo) × 2 (time: baseline/post-drink) mixed-design ANOVA was used to check differences in Urge scores. Significant main effects of time [*F*(1, 140) = 54.66, *p* < 0.001, *η*_p_^2^ = 0.28] and group [*F*(1, 140) = 4.05, *p* = 0.046, *η*_p_^2^ = 0.03] showed urge was greater post-drink and in the alcohol group. Follow-up independent *t*-tests confirmed no difference between groups at baseline [*t*(140) = −0.41, *p* = 0.68, *d* = 0.07], but a significant difference post-drink [*t*(140) = 3.44, *p* = 0.001, *d* = 0.58].

To follow up the group × time interaction [*F*(1, 140) = 23.38, *p* < 0.001, *η*_p_^2^.14], urge change scores were calculated (Urge post-drink – Urge pre-drink) and *t*-test revealed a greater increase in urge in the alcohol, compared to the placebo, group [*t*(140) = 4.83, *p* < 0.001, *d* = 0.81].

Notably, urge change scores in the alcohol group positively correlated with AUDIT scores (*r* = 0.25, *p* < 0.025), TLFB weekly alcohol consumption (*r* = 0.25, *p* < 0.025), and TLFB binge scores (*r* = 0.27, *p* < 0.025). There were no significant correlations between urge change scores and alcohol use indices in the placebo group.

### Behavioural risk taking

Alcohol consumption increased risk taking, as measured by Adjusted average pumps (33.37 ± 11.66), on the BART, relative to placebo (28.68 ± 11.49) [*t*(140) = 2.38, *p* = 0.019, *d* = 0.40]. In order to investigate the role of sex differences in alcohol-induced risk taking we also ran a between subjects ANOVA with group and gender as between subjects factors. This analysis revealed no main effect of gender, or group by gender interaction (*p* > 0.1).

### Predicting drinking habits from BART, when controlling for self-report impulsivity and sensation seeking

In order to investigate the predictive utility of the BART in terms of drinking behaviour, hierarchical regression analyses were conducted in which weekly alcohol consumption was the dependent variable. We ran hierarchical regressions separately for the placebo and alcohol group. In the first step we controlled for impulsivity (BIS total score was moderately associated with weekly alcohol consumption for the entire sample; *r* = 0.16, *p* = 0.06) and sensation seeking (SS correlated with weekly alcohol consumption; *r* = 0.24, *p* = 0.004). Adjusted average pumps were added as the second step. In the placebo group, adjusted average pumps did not predict a significant amount of variance in alcohol use after controlling for BIS and SS (*p* > 0.1). However, in the alcohol group Adjusted average pumps predicted a significant amount of variance in weekly consumption after controlling for BIS and SS [*R* ^2^ = 0.11, *R* ^2^ change = 0.7, *F*-change (1,67) = 5.01, *p* = 0.03; *β* = 0.27].

Identical results were found when this analysis was repeated with weekly binges as the dependent variable. In the placebo group, adjusted average pumps did not predict variance in bingeing after controlling for BIS and SS (*p* > 0.1). In the alcohol group: adjusted average pumps predicted variance in binges after controlling for BIS and SS [*R* ^2^ = 0.07, *R* ^2^ change = 0.04, *F*-change (1,66) = 4.97, *p* = 0.032; *β* = 0.27].

### Does alcohol-induced risk taking mediate alcohol priming (urge to drink)?

Mediation analysis investigated whether BART risk taking accounted for any increase in alcohol urge from baseline to post drink. Joint significance testing method was chosen based on our sample size, effect sizes and recommendations by Mackinnon et al. (2002). We used the joint significance test to assess the relationship between the IV (group) and the mediator (BART; α path), mediator (BART) and DV (Urge changes score; β path)*.* If both paths are significant there is evidence of mediation. Furthermore we used PRODCLIN (MacKinnon et al. 2007) to calculate asymmetrical confidence intervals of this indirect effect. We used this method as it is a more sensitive measure than product of the coefficient tests such as the Sobel test. Indeed, due to the nature of our data (and indeed most data sets on which simple mediation analysis is conducted) the product of the α and β paths would not be normally distributed as well as leptokurtic (see Bollen and Stine 1990; MacKinnon et al. 2002), necessitating the calculation of asymmetrical confidence intervals (see Fritz and MacKinnon 2007; MacKinnon et al. 2007).

The effect of group on BART performance was significant (α path; *r* ^2^ = 0.04, *β* = 0.20, *p* = 0.019) as was the association between BART performance and increased urge (β path; *r* ^2^ = 0.04, *β* = 0.19, *p* = 0.023]. PRODCLIN revealed that the upper and lower 95 % confidence limits for the indirect effect of group on urge increase were >1. This indicates statistically significant mediation of the alcohol priming effect by intoxicated risk taking (as measured by the BART, CL_.95_ 1.53–0.02).

## Discussion

This work, to the best of our knowledge, is the first study to show an acute effect of alcohol on the BART, a behavioural measure of risk taking. Our hypothesis, that alcohol would increase risking taking, relative to placebo, was supported. Regression analysis showed that in the alcohol group only, BART performance predicted a significant proportion of variance within weekly alcohol consumption and binge frequency beyond that of self-reported trait BIS and SS, suggesting that those susceptible to the risk-inducing effects of alcohol may be more likely to drink excessively. Mediation analysis showed that BART performance following alcohol consumption mediated the alcohol priming effect on urge to drink, therefore, this may be one mechanism by which susceptibility to alcohol-induced risk taking is associated with heavier drinking.

The relationships between impulsivity and alcohol use have been well documented (Christiansen et al. 2012a; Leeman et al. 2007; Murphy and Garavan 2011), and evidence shows that alcohol affects specific aspects of impulsivity which may be particularly important in drinking. For example, Weafer and Fillmore (2008) found that alcohol-induced impairment on a Go/No-Go task (measures the inhibitory control aspect of impulsivity) was positively correlated with alcohol consumption during a separate experimental session.

Although risk taking has also been identified as an aspect of impulsivity associated with drinking (Lane et al. 2004), there is a lack of research determining whether alcohol-induced increases in risk taking are predictive of drinking. Uniquely, our study showed that moderate alcohol consumption can increase risk taking on the BART and that this effect predicts drinking behaviour. Our finding, that alcohol increases risk taking on the BART, is in contrast to that of other studies which have assessed alcohol's acute effects on this task (Reynolds et al. 2006; Peacock et al. 2013). These discrepancies may be due to a number of methodological differences: administering different alcohol doses (alcohol doses across studies ranged from 0.4 to 0.8 g/kg, which can affect cognition and behaviour differently); multiple tasks (Reynolds et al. administered several tasks which may have led to fatigue); and differences in blood alcohol levels at time of testing. Although we and Peacock et al. administered the BART 25–30 min post-drink, during ascending blood alcohol levels (BrAC, ~0.36 mg/l) when people feel more stimulated (King et al. 2002), Reynolds et al. tested 15–105 min post-drink which would have led to a large range of BrAC readings and testing during ascending and descending blood alcohol levels. In addition, there were differences in the drinking characteristics of participants; the small number of participants in Reynolds et al.'s (2006) study were light drinkers (6.6 drinks p/week) compared with our moderate to heavy social drinkers (22.6 units p/week). With respect to Peacock et al.'s study, our sample also had higher weekly consumption rates (~184 vs. 145 g) and AUDIT scores (15 vs. 8). Given our finding that alcohol-induced risk taking is predictive of heavier drinking, it is possible that light drinkers are less susceptible to alcohol's effect on risk taking. However, it is important to remember that our sample were non-dependent drinkers, so this data does not allow us to identify whether alcohol-induced risk taking is an important mechanism in dependent drinking. Future research is also needed to determine whether alcohol-induced risk taking and the association between alcohol-induced risk taking and priming is a consequence, or risk factor for, the development of more hazardous drinking.

Although Reynolds et al. (2006) study may have been under-sampled we also acknowledge that our large sample allowed identification of a range of small–large effects. However, it is important to note that our sample were healthy students, and not selected on the basis of risk taking or susceptibility to alcohol-induced risk taking. Future research should identify what makes an individual vulnerable to, or protected against, this susceptibility; it is possible that they have a measurable risk factor for harmful drinking which could be an intervention target.

Weafer and colleagues (2011) found a trend association between (sober) BART and weekly alcohol consumption, and argued that high risk taking individuals may show an enhanced sensitivity to reward and a blunted sensitivity to punishment, which results in excessive drinking. Although this may be the case, our findings differ from Weafer et al. (2011) as we did not find that BART performance predicted drinking behaviour within our placebo group. Given that our regression analysis showed that sober BART performance did not predict any variance in drinking behaviour beyond that of self-reported BIS and SS, it is possible that the trend effects found by Weafer et al. (2011) may have been lost, or reduced, if they had controlled for these personality traits. However, it should be noted that our correlational analysis (which did not take into account trait-like impulsivity) also failed to find an association between sober BART performance and drinking habits (see Supplemental document). It is possible that there was a fundamental difference across our participant samples; Weafer et al. (2011) included participants with ADHD. Although Weafer et al. did not find statistical differences in BART performance between their ADHD and control participants, it is possible that the link between general risk taking and drinking is more pronounced in those with ADHD which is something for future research to explore.

Fernie et al. (2010) also found a relationship between sober BART performance and drinking habits when using an 'alcohol use index' score, comprised of the AUDIT, TLFB and a Binge Drinking questionnaire. It is possible that a more complex index of alcohol behaviour may have shown greater associations with sober BART performance in the current sample and this is something for future research to clarify (please see Supplemental information for further discussion). An alternative possibility involves our inclusion of a placebo group. Previous work has shown that alcohol priming measures differ depending on whether real or placebo alcohol has been consumed (Rose et al. 2013). It is possible that the lack of a pharmacological alcohol effect when alcohol was expected may have somehow disrupted BART performance, resulting in the discrepancies observed between our data and others, and this is something for future research to determine.

Importantly, our results highlight a possible mechanism by which alcohol-induced risk taking may have its effect. Mediation analysis revealed that alcohol priming (urge to drink) was mediated by alcohol-induced increases in risk taking. Most research focuses on how alcohol-induced disinhibition is related to drinking (e.g., ad lib consumption) (Weafer and Fillmore 2008) or how the subjective effects of alcohol, such as stimulation and urge, may be related to drinking (King et al. 2011). There has been no research, to our knowledge, which has tested whether urge-related priming effects are related to alcohol-induced risk taking. Rose and Grunsell (2008) found that naturally impulsive individuals showed a greater alcohol priming effect, but they did not test whether intoxicated performance on a task of inhibitory control (Go/No-Go) was related to priming. This is an important area of research; our findings show that the priming effect measured in the laboratory is positively correlated with drinking indices therefore, understanding the mechanism underlying priming should help clarify the basis of hazardous drinking.

Our findings may be in line with the theoretical proposal that alcohol acutely impairs inhibitory control which increases drinking both directly and by allowing appetitive alcohol responses to continue unchecked (Field et al. 2010). It is possible that increases in a range of aspects of impulsivity, including risk taking, may impair our ability to inhibit appetitive responses. Alternatively, as risk takers tend to be those willing to engage in behaviours based on potential positive consequences relative to negative consequences (MacPherson et al. 2010), alcohol-induced risk taking may somehow increase the incentive salience of alcohol's positive effects, which then results in a greater urge to drink. It is important for future research to identify the mechanisms by which risk taking has its effect on priming factors.

It is interesting to note that the lack of research linking aspects of impulsivity and craving has been recently highlighted by others (Joos et al. 2013), and that research is beginning to emerge which shows that these two mechanisms of drinking may be associated. For example, greater delay discounting (MacKillop et al. 2010) and reduced reflection impulsivity (tendency to gather and evaluate information before making a decision) correlates with craving (Joos et al. 2013), while impaired response inhibition (stop signal task) moderates alcohol cue-induced craving (Papachristou et al. 2012). The current paper is the first to investigate associations between alcohol-induced risk taking and priming but we would suggest that this is a fruitful area of research.

In terms of current limitations and recommendations for future research, we would highlight several points. Firstly, real rewards should be used as it is possible that participants will act in a more risky way with rewards they know are hypothetical as they are not as invested in the final outcome. Although the use of hypothetical rewards does not diminish our findings that drinking behaviour is associated with risk taking, future research which uses real rewards can make more definitive claims regarding the real world importance of such evidence. Secondly, we would advise using a within subject design. Given previous findings that adolescent risk taking can decrease across blocks (Lejuez et al. 2005, 2007) we were cautious and used a between-subjects design. We are confident that our findings are not due to pre-existing differences (our participants were taken from the same population and there were no differences in participant characteristics across our two groups) but future within-subjects research will provide stronger evidence.

Although our data needs to be confirmed with follow-up research, this study indicates that the BART is a useful behavioural measure for investigating the acute effects of alcohol on risk taking. Importantly, results suggest that individuals susceptible to alcohol-induced risk taking may drink more heavily, and that this risk may partly work through alcohol priming effects.

## Electronic supplementary material

Below is the link to the electronic supplementary material.

ESM 1 (DOCX 28 kb)
