# Supplementary material for: Alcohol-induced risk taking on the BART mediates alcohol priming
Source: Psychopharmacology (Berl). 2013 Dec 13;231(11):2273–80. doi: 10.1007/s00213-013-3377-1 (PMC4018511; doi:10.1007/s00213-013-3377-1)
Supplement: Supplementary file 1 — (DOCX 28 kb) [file 213_2013_3377_MOESM1_ESM.docx]

**Supplemental Information**

Given that research has found different relationships between aspects of impulsivity (e.g., self-report BIS, SS, and behavioural BART), and between aspects of impulsivity and drinking behaviour, we also conducted correlational analysis to clarify these relationships in our alcohol and placebo conditions.

**Results**

*Correlations between BART and measures of alcohol consumption (see Table 1)*

Table one demonstrates that the associations between alcohol use indices and BART performance differed across groups. Risk taking following moderate alcohol consumption correlated with both weekly unit consumption [p= .001] and binge frequency [p = .006], but not AUDIT. However, BART performance did not correlate with any drinking habit measures in the placebo group. Further, we investigated whether the magnitude of the correlation coefficients differed across groups using the method described by Preacher (2002). The only significant difference in the magnitude of correlations was between Adjusted average pumps and weekly alcohol consumption [p = .036].

In order to investigate whether there were any sex differences in the association between BART performance and risk taking in either group, a partial correlation was conducted in which we controlled for gender (Table 1). Controlling for gender did not significantly affect any of the associations, suggesting there were no gender-specific effects.

Insert Table 1

*Correlations between BART and self-report impulsivity and sensation seeking (see Table 2)*

Table two demonstrates that BART performance following alcohol consumption was not associated with either self-report impulsivity or sensation seeking. Following placebo, Adjusted average pumps, correlated with sensation seeking [p=.028].

Insert Table 2

Comment

Previous research has shown that risk taking on the BART is associated with higher levels of alcohol and substance use ([Fernie et al., 2010](#_ENREF_2); [Lejuez et al., 2003](#_ENREF_3); [Lejuez et al., 2002](#_ENREF_4)). However, our placebo data failed to find any correlation between BART and drinking habits. Although Lejuez et al’s (2002) sample was taken from college campuses and the community, drinking habits were not reported so it is possible that discrepancies arise from differences in participant characteristics. Fernie and colleague’s (2010) sample was taken from the same university population as the current study, however, their drinking habits were calculated as an ‘alcohol use index’ score which included data from the AUDIT, TLFB and a Binge Drinking questionnaire. It is possible that a more complex index of alcohol behaviour may have shown greater associations with BART performance in the current sample and this is something for future research to clarify. However, the positive correlation between BART and weekly consumption and binge frequency in our alcohol condition does fit with the findings from our regression and mediation analysis; alcohol-induced risk taking predicts drinking behaviour, and this may be at least partly because increases in risk taking following alcohol consumption mediates increases in priming measures (which are associated with increased drinking levels) ([Rose & Grunsell, 2008](#_ENREF_6)).

Within the existing literature, there are discrepancies in the association between self-reported and behavioural impulsivity. In adults, Lejuez et al. (2002) found a relationship between the BART and the BIS, however, research on adolescents has failed to find this association ([Aklin et al., 2005](#_ENREF_1); [Lejuez, et al., 2003](#_ENREF_3)). Our results are similar to that of Reynolds et al., ([2006](#_ENREF_5)) who found no relationship between the BART and BIS. The indication that some self-report and behavioural measures are tapping different underlying constructs is not surprising; the BIS measures stable aspects of impulsivity along dimensions of motor control, cognitive control, and lack of planning ([Stanford et al., 2009](#_ENREF_7)), while the BART is a behavioural state measure of our willingness to risk loss for the chance of higher gain. We did, however, find a correlation between BART and self-reported sensation seeking when sober (although not when intoxicated, indicating that trait-like sensation seeking is not associated with alcohol’s ability to increase risk taking). Although impulsivity and sensation seeking are often used together, sensation seeking illustrates a specific component of impulsivity; it is a tendency to seek out novel, varied, and intense experiences, and the willingness to take risks for such experiences ([Zuckerman & Kuhlman, 2000](#_ENREF_8)). It is arguable therefore, that risk taking will be more influenced by sensation seeking, relative to more general impulsive characteristics. It is interesting that Reynolds et al., (2006) found that different behavioural tasks also measure specific aspects of impulsivity; the BART and Delay Discounting tasks fell into a category of ‘impulsive decision making’, while the Go/No-Go and Stop tasks fell into a category of ‘impulsive disinhibition’. This highlights both the need to identify which components of impulsivity influence drinking, and to ensure that research selects appropriate combinations of self-report and behavioural task.

**References**

Aklin WM, Lejuez CW, Zvolensky MJ, Kahler CW, Gwadz M (2005) Evaluation of behavioral measures of risk taking propensity with inner city adolescents Behavior Research Therapy 43: 215-228

Fernie G, Cole JC, Goudie AJ, Field M (2010) Risk-Taking but Not Response Inhibition or Delay Discounting Predict Alcohol Consumption in Social Drinkers. Drug and Alcohol Dependence 112:54-61

Lejuez CW, Aklin WM, Zvolensky MJ, Pedulla CM (2003) Evaluation of the Balloon Analogue Risk Task (BART) as a predictor of adolescent real-world risk-taking behaviours. Journal of Adolescence 26: 475-479

Lejuez CW, Read JP, Kahler CW, Richards JB, Ramsey SE, Stuart GL, Brown RA, et al. (2002) Evaluation of a behavioral measure of risk taking: The Balloon Analogue Risk Task (BART). Journal of Experimental Psychology: Applied 8: 75-84

Preacher KJ (2002, May) Calculation for the test of the difference between two independent correlation coefficients [Computer software]. Available from [http://quantpsy.org](http://quantpsy.org/)

Reynolds B, Ortengren A, Richards JB, de Wit H (2006) Dimensions of impulsive behavior: Personality and behavioral measures. Personality and Individual Differences 40: 305-315

Rose AK, Grunsell L (2008). The subjective, rather than the disinhibiting, effects of alcohol are related to binge drinking. Alcoholism Clinical and Experimental Research 32: 1096-1104

Stanford MS, Mathias CW, Dougherty DM, Lake SL, Anderson NE, Patton JH (2009) Fifty years of the Barratt Impulsiveness Scale: An update and review. Personality and Individual Differences 47: 385-395

Zuckerman M, Kuhlman DM (2000) Personality and risk-taking: Common biosocial factors. Journal of Personality 68: 999-1029

Table 1: Pearson’s correlations between alcohol use indices and BART Adjusted average pumps, shown separately for alcohol and control conditions (**p<.01; *p<.025).

Alcohol Placebo

BART Measure AUDIT TLFB Total TLFB Binge AUDIT TLFB Total TLFB Binge

Adjusted Average Pumps .17 .36**† .30** .18 .07 .12

Controlling for gender

Adjusted Average Pumps .17 .33** .29* .19 .09 .12

AUDIT = Alcohol use disorders identification test, possible range of scores is from 0 (minimum) to 40 (maximum). TLFB Total = Past 14 day alcohol consumption in UK units, 1 unit = 8g alcohol; TLFB Binge = number of binges per week, binges defined as ≥6 drinks (females) or ≥8 drinks (males) in a single day. Adjusted average pumps = average number of pumps on unexploded balloon trials.

† Denotes significant difference in the magnitude of correlations between conditions

Table 2: Pearson’s correlations between self-report measures of impulsivity and sensation seeking and BART outcome measures, shown separately for alcohol and control conditions (*p<.05).

Alcohol Placebo

BART Measure BIS Total Sensation Seeking BIS Total Sensation Seeking

Adjusted Average Pumps -.11 .08 .15 .23*

BIS Total = Barratt Impulsivity scale BIS total score (higher values indicate greater impulsivity). Sensation Seeking = total score on sensation seeking subscale of Zuckerman–Kuhlman Personality Questionnaire (higher values indicate greater sensation seeking). Adjusted average pumps = average number of pumps on unexploded balloon trials.
